# Supplementary material for: Synthesis of FeOOH-Loaded Aminated Polyacrylonitrile Fiber for Simultaneous Removal of Phenylphosphonic Acid and Phosphate from Aqueous Solution
Source: Polymers (Basel). 2023 Apr 17;15(8):1918. doi: 10.3390/polym15081918 (PMC10146033; doi:10.3390/polym15081918)
Supplement: Supplementary file 1 [file polymers-15-01918-s001.zip › polymers-2328238-SM.pdf]

Supporting Information

Synthesis of FeOOH-Loaded Aminated Polyacrylonitrile Fiber for Simultaneous Removal of Phenylphosphonic Acid and Phosphate from Aqueous Solution

Rui Zhou [1,2,†], Wusong Xu [1,2,†], Peisen Liu [1,2], Shangyuan Zhao [1,2], Gang Xu [1,2,*], Qizhong Xiong [1,2], Weifeng Zhang [1,2], Chaochun Zhang [1,2] and Xinxin Ye [1,2]

[1] Anhui Province Key Lab of Farmland Ecological Conservation and Pollution Prevention, Engineering and Technology Research Center of Intelligent Manufacture and Efficient Utilization of Green Phosphorus Fertilizer of Anhui Province, College of Resources and Environment, Anhui Agricultural University, Hefei 230036, China

[2] Key Laboratory of JiangHuai Arable Land Resources Protection and Eco-Restoration, Ministry of Natural Resources, College of Resources and Environment, Anhui Agricultural University, Hefei 230036, China

* Correspondence: gangxu@ahau.edu.cn

† These authors contributed equally to this work.

S1. Materials and reagents

HCl， HNO_3 and H_2SO_4 were purchased from Xilong Scientific Co., Ltd. Ammonium molybdate, *p*-chloronitrobenzene, ascorbic acid and $Na_2S_2O_8$, KH_2PO_4 were purchased from Shanghai Aladdin Biochemical Technology Co., Ltd. Antimonyl potassium tartrate was obtained by Saan Chemical Technology Co., Ltd. Methyl alcohol. Methyl alcohol Shanghai Macklin Biochemical Co., Ltd. Na_2CO_3, $NaHCO_3$, NaOH and K_2CO_3 were supplied from Shanghai SuYi Chemical Reagent Co., Ltd. K_2SO_4, KCl and NaCl were provided by Sinopharm Group Chemical Reagent Co., Ltd. Methanol is chromatography-grade pure. The water used in this experiment is deionized water and chemicals used in this study are analytical grade unless otherwise specified.

S2. Characterization

The surface morphology and element distribution of the fibers were determined by scanning electron microscope (S-4800, Hitachi) and X-ray energy dispersive spectrometer (EDS, X-Max N 150). The changes of functional groups and elemental content (C, N and O) on the fiber surface were measured by Fourier Infrared Spectrometer (Nicolette is50, USA) and elemental analyzer (Vario EL Cube, Germany), respectively. The test method of Fourier Infrared chromatography is to cut fiber into pieces, mix the fiber with potassium bromide, and press them into thin sheets, then measure it in the wavenumber range of 4000-400 cm^{-1}. The internal crystal structure of different fibers is performed by the D/MAX-2500 X-ray diffraction (XRD) (the Confucian Co., Ltd.) within the range of 10°-90° at a speed of 4 ° per minute. Thermal stability of the fibers was tested by a STA409F5TGA/DSC simultaneous thermal analyzer (Netzsch company, Germany) from room temperature to 800 °C at a rate of 10 °C min^{-1} in nitrogen atmosphere. The surface structure and elemental composition of the fibers were characterized by X-ray photoelectron spectroscopy (XPS, PERKIN ELMZR, USA), Full-spectrum scan flux energy is 150 eV in 1 eV steps; narrow-spectrum scan flux energy is 50 eV in 0.1 eV steps. The species of free radicals in the reaction system were determined by Electron paramagnetic resonance spectrometer (EPR, Bruker EMXplus, Germany) at the 5th min from the beginning of the reaction.

S3.Steps and methods for the determination of PPOA and phosphate

PPOA: After the degradation experiment, the fibers were separated from the solution with tweezers, 1 mL of sample solution was filtered by 0.22 μm filter membrane, and 1 mL of methanol was added immediately for quenching. The remaining PPOA content was then determined by HPLC. The set conditions of the HPLC are: methanol as a mobile

phase, methanol to water ratio of 4:1, flow rate of 0.8, wavelength of 220 nm, the use of BaseLine C18 liquid chromatography column.

Phosphate: After the adsorption test, the fibers were separated from the solution with forceps, and 1 mL of the sample solution was taken into a volumetric flask and the remaining phosphate content was determined in a spectrophotometer using the molybdenum blue colorimetric method. The wavelength of the spectrophotometer was used at 700 nm.

S4. Reusability experiments

S4.1 The step of degradation cycle by PANAF-FeOOH

To investigate the cycling performance of PANAF-FeOOH. After 30 mg PANAF-FeOOH was added to a 100 mL glass vial containing 5 mg L^{-1} of PPOA and 0.5 mmol L^{-1} PDS at 25 °C for the reaction. When the reaction is over, the material was recovered using forceps and washed repeatedly with deionized water, then the fiber undergoes the next repeated reaction. The above process was repeated successively to test the resuability ability of PANAF-FeOOH.

S4.2 The step of adsorption cycle by PANAF-FeOOH

After weighing 50 mg of dried PANAF-FeOOH into 20 mL of KH_2PO_4 solution with an initial phosphate concentration of 2 mg P L^{-1} and stirring for 1 h, the phosphate concentration in the solution was measured by removing the fibers with forceps. The fiber was rinsed repeatedly with deionized water to clean the phosphate adhering to the surface, then the fiber transferred to 20 mL of sodium hydroxide solution with an initial concentration of 0.05 mol L^{-1} for desorption for 1 h. The phosphate concentration in the solution was measured. Then the next adsorption experiment was conducted directly after the fiber is washed.

S5. Synthesis and optimization of PANAF-FeOOH

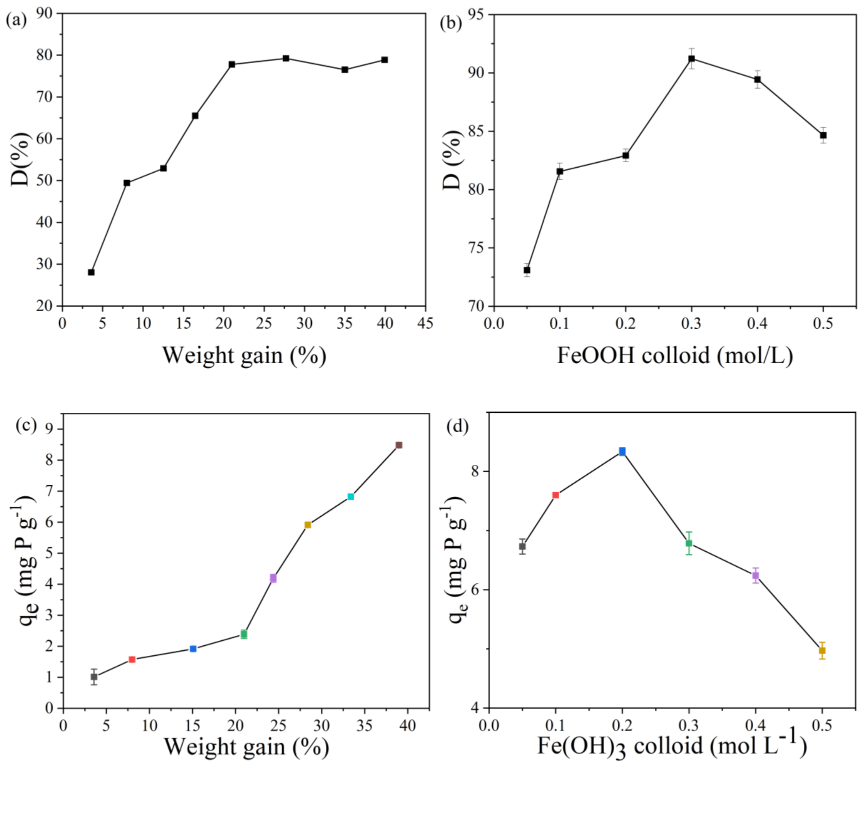

Figure S1. (a) The degradation effect of PPOA by 0.3 mol/L $Fe(OH)_3$ combined with aminated fibers with different weight gain; (b) The degradation effect of PPOA by aminated fibers with weight gain

of 38% combined with different concentrations of $Fe(OH)_3$ colloid; (c) Phosphate adsorption capacity of 0.2 mol/L $Fe(OH)_3$ combined with aminated fibers with different weight gain; (d) Phosphate adsorption capacity of aminated fibers with weight gain of 38% combined with different concentrations of $Fe(OH)_3$ colloid.

S6. Adsorption kinetics

Table S1. Kinetic parameters of phosphate adsorption by PANAF-FeOOH.

T (K)	Pseudo-first-order kinetic model			Pseudo-second-order kinetic model		
	K_1 (min^{-1})	q_e (mg P g^{-1})	R^2	K_2 (g mg^{-1} min^{-1})	q_e (mg P g^{-1})	R^2
288	0.485	7.91	0.907	0.094	8.38	0.997
298	0.575	8.27	0.872	0.105	8.76	0.983
308	0.607	8.79	0.850	0.108	9.28	0.992

S7. Adsorption thermodynamics

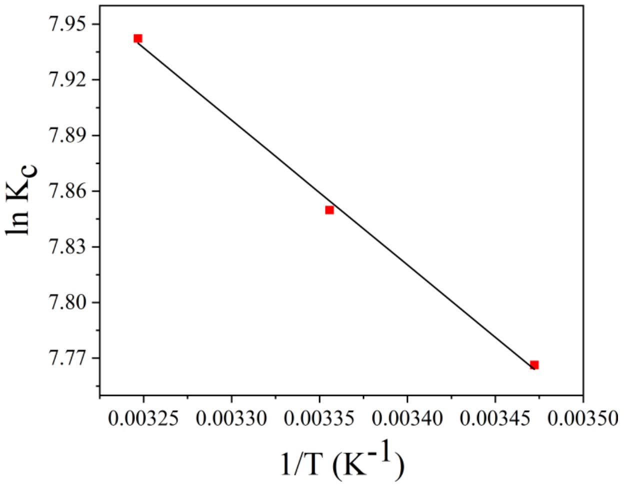

Figure S2. Fitting diagram of thermodynamics of phosphate.

Table S2. Thermodynamic parameters of phosphate adsorption by PANAF-FeOOH.

T (K)	G° (kJ mol^{-1})	ΔH° (kJ mol^{-1})	ΔS° (J mol^{-1} K^{-1})
288	-18.596		
298	-19.448	6.482	87.056
308	-20.338		

S8. Adsorption isotherm

Table S3. Isotherm parameters of phosphate adsorption by PANAF-FeOOH.

Langmuir			Freundlich		
q_e (mg P g^{-1})	KL (L mg^{-1})	R^2	K_F [mg/g·(L/mg)$^{1/n}$]	1/n	R^2
9.918	0.281	0.990	2.954	0.330	0.921

S9. Continuous flow experiment

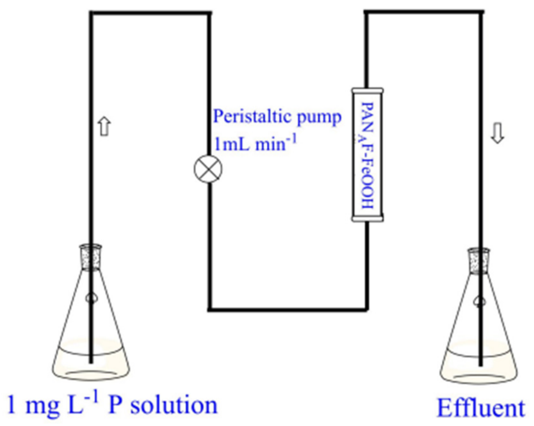

Figure S3. Simple diagram of continuous flow process device.

S10. EPR

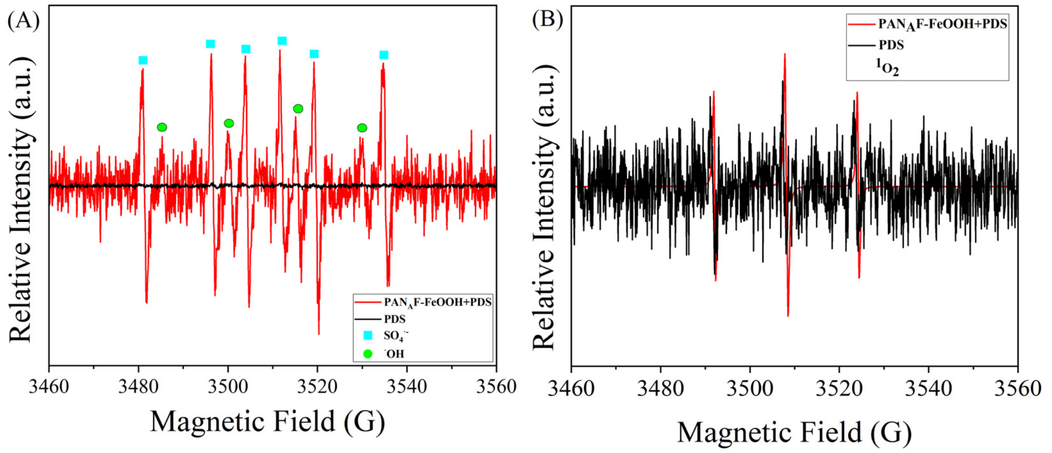

Figure S4. (A) EPR spectrum of SO_4^{-}• and •OH of PDS/PDS- PAN$_A$F-FeOOH atalytic system; (B) EPR spectrum of $^1O_2$ of PDS/PDS- PAN$_A$F-FeOOH atalytic system.

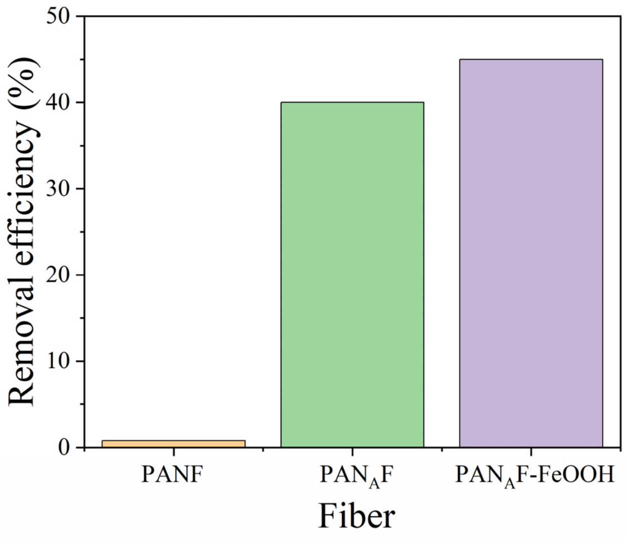

Figure S5. Adsorption capacity of PAN$_A$F-FeOOH for PPOA (30 mg of PAN$_A$F-FeOOH was soaked in 50 mL of PPOA with concentration of 5 mg P L^{-1} for 2 h, pH=5).

S11. XPS

Table S4. The mass and atomic percentage of the functionalized fiber obtained by XPS.

Fiber	C	N	O	Fe	P
PANF	73.78	7.71	18.51	0	0
PAN_AF	79.55	10.14	10.31	0	0
PAN_AF-Fe	46.34	8.65	36.82	8.2	0
PAN_AF-Fe-P	57.67	3.13	30.44	6.01	2.76

S12.Comparison of different adsorbents

Table S5. Comparison of different phosphate adsorbents.

Number	Materials	q_m (mg P g^{-1})	Run	Adsorption equilibrium	References
1	FeOOH-GO	5.81	/	7 h	[1]
2	Amorphous FeOOH	98.69	/	4 h	[2]
3	FeOOH@MS	115.5	/	2 h	[3]
4	FeOOH/BF	39.08	3	12 h	[4]
5	FSP-f	7.6	3	48 h	[5]
6	β-FeOOH/GO	45.2	/	120 min	[6]
7	α-FeOOH-600BC	11.39	/	192 h	[7]
8	PAN_AF-FeOOH	9.92	5	30 min	This manuscript

References

1. Li, X.; Elgarhy, A.H.; Hassan, M.E.; Chen, Y.; Liu, G.; Elkorashey, R.M. Removal of inorganic and organic phosphorus compounds from aqueous solution by ferrihydrite decoration onto graphene. *Environ. Monit. Assess.* **2020**, *192*, 1–9, https://doi.org/10.1007/s10661-020-08325-y.
2. Zhang, X.; Yao, H.; Lei, X.; Lian, Q.; Roy, A.; Doucet, D.; Yan, H.; Zappi, M.E.; Gang, D.D. A comparative study for phosphate adsorption on amorphous FeOOH and goethite (α-FeOOH): An investigation of relationship between the surface chemistry and structure. *Environ. Res.* **2021**, *199*, 111223, https://doi.org/10.1016/j.envres.2021.111223.
3. Tao, R.; Qu, M.; Zhang, S.; Quan, F.; Zhang, M.; Shen, W.; Mei, Y. Preparation of FeOOH supported by melamine sponge and its application for efficient phosphate removal. *J. Environ. Chem. Eng* **2022**, *10*, https://doi.org/10.1016/j.jece.2022.108064.
4. Ai, H. Y.; Li, X. H.; Chen, C.; Xu, L.; Fu, M. L.; Sun, W. J.; Yuan, B. L., Immobilization of β-FeOOH nanomaterials on the basalt fiber as a novel porous composite to effectively remove phosphate from aqueous solution. *Colloid. Surface. A.* 2022, *632*, *127815.*
5. Wu, Z.-Y.; Zhao, H.; Hu, X.; Yuan, B.; Fu, M.-L. Tunable porous ferric composite for effective removal of phosphate in water. *Colloids Surfaces A: Physicochem. Eng. Asp.* **2019**, *582*, 123854, https://doi.org/10.1016/j.colsurfa.2019.123854.
6. Harijan, D.K.; Chandra, V. Akaganeite nanorods decorated graphene oxide sheets for removal and recovery of aqueous phosphate. *J. Water Process. Eng.* **2017**, *19*, 120–125, https://doi.org/10.1016/j.jwpe.2017.07.019.
7. Zhang, X.; Gang, D.D.; Sun, P.; Lian, Q.; Yao, H. Goethite dispersed corn straw-derived biochar for phosphate recovery from synthetic urine and its potential as a slow-release fertilizer. *Chemosphere* **2020**, *262*, 127861, https://doi.org/10.1016/j.chemosphere.2020.127861.
